# Improved Recurrence Plots Compression Distance by Learning Parameter for Video Compression Quality

**DOI:** 10.3390/e25060953

**Published:** 2023-06-19

**Authors:** Tatsumasa Murai, Hisashi Koga

**Affiliations:** Department of Computer and Network Engineering, University of Electro-Communications, Tokyo 182-8585, Japan

**Keywords:** time series classification, compression-based pattern recognition, data compression, MPEG-1, recurrence plots

## Abstract

As the Internet-of-Things is deployed widely, many time-series data are generated everyday. Thus, classifying time-series automatically has become important. Compression-based pattern recognition has attracted attention, because it can analyze various data universally with few model parameters. RPCD (Recurrent Plots Compression Distance) is known as a compression-based time-series classification method. First, RPCD transforms time-series data into an image called “Recurrent Plots (RP)”. Then, the distance between two time-series data is determined as the dissimilarity between their RPs. Here, the dissimilarity between two images is computed from the file size, when an MPEG-1 encoder compresses the video, which serializes the two images in order. In this paper, by analyzing the RPCD, we give an important insight that the quality parameter for the MPEG-1 encoding that controls the resolution of compressed videos influences the classification performance very much. We also show that the optimal parameter value depends extremely on the dataset to be classified: Interestingly, the optimal value for one dataset can make the RPCD fall behind a naive random classifier for another dataset. Supported by these insights, we propose an improved version of RPCD named qRPCD, which searches the optimal parameter value by means of cross-validation. Experimentally, qRPCD works superiorly to the original RPCD by about 4% in terms of classification accuracy.

## 1. Introduction

As theInternet-of-Things (IoT) prevails, a lot of time-series data are emitted from sensors in daily life. Thus, classifying time-series data automatically has become an important topic. While Deep Neural Networks (DNNs) achieve high classification accuracy, they must usually learn an enormous amount of training data over a long time period. Furthermore, DNNs tend to be task-specific, so that they must be trained newly to be applied to another classification task.

By contrast, compression-based pattern recognition involves very few parameters. Thus, it is often regarded as a universal method that can analyze various kinds of data such as time series, genome, and music at low cost. Typically, this approach obtains the similarity between two objects *x* and *y* from the file size after compressing the concatenation of *x* and *y*. NCD (Normalized Compression Distance) [1] is the most well-known compression distance between two objects. Although lossless compression algorithms like LZW and bzip2 are utilized in most compression distances, they favor one-dimensional strings and are not suitable for handling images that have a two-dimensional structure. So, this restriction forces us to convert an image into a string by some means [2].

To escape from this constraint, Campana et al. [3] devised a CK-1 distance (Campana–Keogh distance) between two images *x* and *y* founded upon a lossy compression method MPEG [4]. It first makes a two-frame video that combines *x* with *y* then compresses the video with the MPEG-1 encoder. The CK-1 distance is obtained from the file size of the compressed video.

Silva et al. [5] applied the CK-1 distance to time-series classification, which is called the Recurrence Plots Compression Distance (RPCD). Here, time-series data are represented as an image called Recurrence Plots (RP). The RPCD defines the distance between two time-series data as the CK-1 distance between their RPs. The greatest merit of RPCD is the ease of deployment: It is claimed in [5] that non-experts in pattern recognition can easily compute the RPCD, only if they have an MPEG-1 encoder publicly available.

We believe that the compression distance like RPCD will continue to be important in the future, because it allows us to analyze data with only a few model parameters. For instance, it is very laborious to model unknown time-series data emitted from new sensors with many parameters. However, the inventor of RPCD does not describe how to operate the MPEG-1 encoding in detail. Thus, in order to raise the classification accuracy of the RPCD, we first access the feature of RPCD by implementing it by ourselves with FFmpeg [6]. As a result, we acquire an important insight that the quality parameter for the MPEG-1 encoding which adjusts the resolution of compressed videos affects the classification accuracy very much. Significantly, the optimal parameter value changes drastically per dataset, such that the optimal value for some dataset can make the RPCD inferior to a naive random classifier for another dataset. Since our analysis did not presume time-series data, our insights will be valid also for the CK-1 distance that is more primitive than the RPCD and that has many applications such as the concept drift detection [7].

The above result also indicates that there exists no parameter value that universally handles any dataset well. Following this observation, we propose an improved version of RPCD named qRPCD that decides the optimal parameter value by learning the training data with cross validation. Experimentally, qRPCD outperformed two previous compression distances based on the RP including the original RPCD by about 4% in terms of classification accuracy.

This paper is organized as follows. Section 2 reviews the MPEG-1 encoding, the RPCD and the previous time-series classification methods that rely on RPs. Section 3 analyzes the RPCD and studies how the quality parameter for MPEG-1 affects the classification accuracy. Section 4 describes our proposed method qRPCD. Section 5 reports the experimental comparison with two previous recurrence plots compression distances. Section 6 concludes this paper.

## 2. Literature Review: MPEG-1 and Recurrence Plots Compression Distance (RPCD)

This section first explains the MPEG-1 encoding briefly, just enough to understand the behavior of the RPCD [5]. Section 2.3 mentions the Cross Recurrence Plots Compression Distance (CRPCD) [8], which is an extension of RPCD. Section 2.4 summarizes previous time-series classification methods that count on the RPs.

### 2.1. Mpeg-1

MPEG-1 is a video compression technique standardized as ISO/IEC 11172. The two key components in MPEG-1 are (1) the intra-frame compression and (2) the motion compensated inter-frame prediction. Whereas the former completes within a single frame, the latter involves multiple frames. Hereafter, we abbreviate the motion compensated inter-frame prediction simply as the inter-frame prediction. The inter-frame prediction compresses a target frame by predicting its pixel values from those in its neighbor frames. MPEG-1 categorizes every frame into one of the three types: I picture, P picture, and B picture. An I picture (Intra-coded picture) is compressed by the intra-frame compression completely without the inter-frame prediction. Therefore, I pictures are decoded without referring to other frames. Next, the pixel values in a P picture (Predicted picture) are predicted from some preceding frame that is either an I picture or a P picture. A B picture (Bi-directional predicted picture) may utilize not only a preceding frame, but also a succeeding frame for the inter-frame prediction. Since the RPCD creates two-frame videos, the B picture never appears in the RPCD.

After converting the pixel colors from RGB to YCbCr, MPEG-1 divides a video frame into macroblocks that are subframes of 16 × 16 size. A macroblock stores a 16 × 16 matrix for the Y value and two sub-sampled 8 × 8 matrices responsible for the Cb and Cr values. More precisely, it consists of four blocks for the Y value, one block for the Cb value and one block for the Cr value, where a block is expressed as an 8 × 8 matrix. MPEG-1 treats a macroblock as a unit to be compressed. MPEG-1 alters how to compress a macroblock according to the type of frame: In an I picture, every macroblock is compressed by the intra-frame compression. On the other hand, macroblocks in a P picture are mostly to be compressed by the inter-frame prediction.

#### 2.1.1. Intra-Frame Compression

In the beginning, the intra-frame compression rewrites any 8 × 8 block in a macroblock from the spatial domain to the frequency domain by the Discrete Cosine Transform (DCT) as shown in Equation (1). For 0≤u,v≤7, F(u,v) is called a DCT coefficient. Especially, F(0,0) represents the average intensity and is called the DC coefficient. The remaining 63 coefficients are termed AC coefficients. One AC coefficient represents the strength of one frequency component. As *u* and *v* become larger, F(u,v) corresponds to a higher frequency component. That is, low-frequency components are stored in the top-left area of the block, while high-frequency components are saved in the bottom-right area.

(1) $$\begin{array}{c} \begin{array}{cc} F(u,v) & =\frac{1}{4}C\left(u\right)C\left(v\right)\sum_{i=0}^{7}\sum_{j=0}^{7}\cos\left(\frac{2i+1}{16}u\pi \right)\cos\left(\frac{2j+1}{16}v\pi \right)\\ \; & C\left(u\right),C\left(v\right)=\left\{\begin{array}{cc} \frac{1}{\sqrt{2}} & \left(u,v=0\right)\\ 1 & \left(u,v\neq 0\right) \end{array}\right. \end{array} \end{array}$$

**Figure 1:**
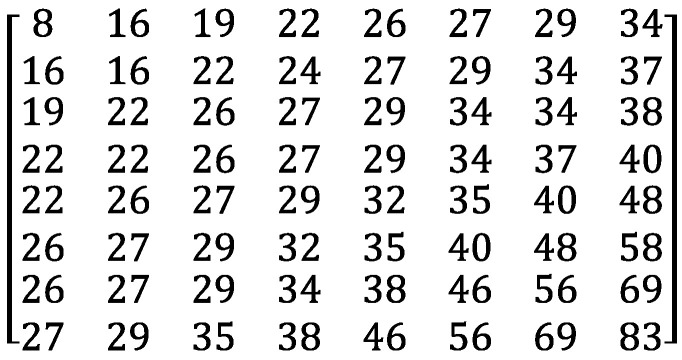
Intra Quantization Matrix $QM_{intra}$.

MPEG-1 assumes that the low-frequency components preserve the important semantics and the high-frequency components hold minute information only that may be noise occasionally. Hence, MPEG-1 realizes high-quality compression by discarding the high-frequency components in *F* by means of *quantization*. The quantization proceeds by dividing each AC coefficient F(u,v) by the intra quantization matrix $QM_{intra}$ in Figure 1 in an element-wise manner. The quantization modifies F(u,v) to $F^{\prime }\left(u,v\right)$ as in Equation (2).

(2) $$F^{\prime }\left(u,v\right)=\mathrm{round}\left(\frac{1}{2qs}\times \frac{F(u,v)}{QM_{intra}\left(u,v\right)}\right).$$

Note that, thanks to $QM_{intra}$, high-frequency components are quantized to 0 with a high probability, because $QM_{intra}$ divides them by large arithmetic values. In Equation (2), qs symbolizes a *quantization scale* parameter. The range of qs consists of integers between 1 and 31. qs controls the extent of video compression. As qs increases, more frequency components reduce to 0 and the frame will be compressed more aggressively.

**Figure 2:**
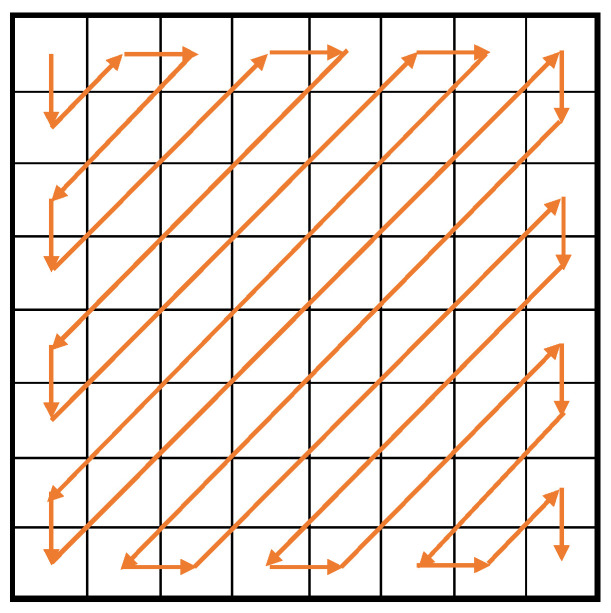
Zig-zag scanning.

Let $F^{\prime }$ be a new block that quantizes *F* with $QM_{intra}$. In $F^{\prime }$, many elements are likely to grow 0 around the bottom-right corner. Hence, MPEG-1 scans $F^{\prime }$ in the zig-zag order in Figure 2 to generate a sequence of figures that are efficiently compressible. Finally, it encodes the figure sequence with the Variable Length Coding (VLC).

#### 2.1.2. Inter-Frame Prediction

Usually, two consecutive frames in a video are quite similar. Therefore, on the condition that the first frame has been already stored, it may be possible to reduce the file size more by remembering the difference between the two frames only than by keeping the whole second frame. The inter-frame prediction embodies this idea.

Suppose that the first frame $f_{1}$ is an I picture and the second frame $f_{2}$ is a P picture. The inter-frame prediction compresses a macroblock mb in $f_{2}$ by searching the most similar macroblock $mb^{\prime }$ in $f_{1}$, where the similarity is determined from the Y (luminance) blocks only. In case $mb^{\prime }$ is too dissimilar to mb, MPEG-1 compresses mb with the intra-frame compression. Otherwise, it records the spatial displacement between mb and $mb^{\prime }$ as a motion vector and their difference in contents as the prediction error. The prediction error is determined individually for the 6 blocks (4 Y blocks, 1 Cr block and 1 Cb block) in a macroblock. For a block, an 8 × 8 matrix expresses the prediction error. Thus, the inter-frame prediction compresses the prediction error by applying the DCT and the quantization in the same way as the intra-frame compression.

**Figure 3:**
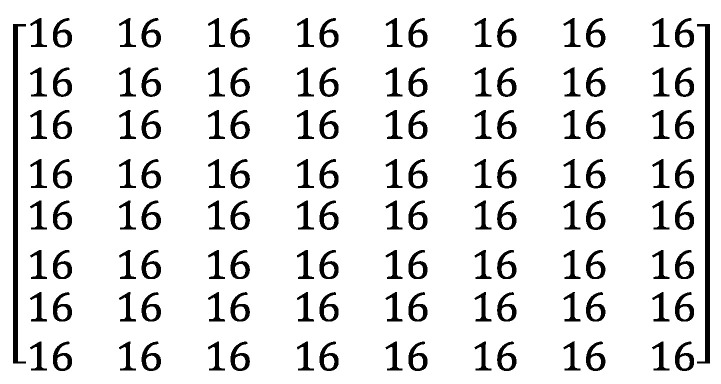
Inter-frame Quantization Matrix $QM_{inter}$.

However, the quantization matrix is quite different. See $QM_{inter}$ in Figure 3. After applying the DCT to the prediction error, all the 64 DCT coefficients that cover all the frequency components usually become small. Therefore, $QM_{inter}$ divides every frequency component evenly without bias. After the quantization, as mb and $mb^{\prime }$ become more similar, more frequency components reduce to 0 and mb will be compressed more compactly.

### 2.2. Recurrence Plots Compression Distance (RPCD)

Let us explain the Recurrence Plots Compression Distance (RPCD). We start by explaining the recurrence plots and the CK-1 distance in Section 2.2.1 and Section 2.2.2, both of which play important roles in the RPCD.

#### 2.2.1. Recurrence Plots

Let *x* be a time-series data. We denote the length of *x* by *N*. Usually, a time-series data contains various recurrent patterns, e.g., seasonality and duly recurrence. Recurrence Plots (RP) [9] is an image that visualizes the recurrent patterns in *x*.

The concept behind RP is very simple. Equation (3) defines the RP for *x*. Here, $\vec{x}\left(i\right)$ presents the *i*-th sub-sequences of *x* whose length equals *m*. $\vec{x}\left(i\right)$ is represented as an *m*-dimensional vector.

(3) $$\begin{array}{c} {\mathrm{RP}}_{i,j}=\Theta (\epsilon -||\vec{x}\left(i\right)-\vec{x}\left(j\right)||),\;\;\;\vec{x}\left(\cdot \right)\in R^{m},1\leq i,j\leq N \end{array}$$

In Equation (3), ϵ is a threshold for closeness and Θ(·) is the step function. In short, the pixel ${\mathrm{RP}}_{i,j}$ becomes black if $\vec{x}\left(i\right)$ and $\vec{x}\left(j\right)$ are similar and $\left|\right|\vec{x}\left(i\right)-\vec{x}\left(j\right)\left|\right|\leq \epsilon$. Otherwise, it becomes white. In this way, the original RP is a binary image.

**Figure 4:**
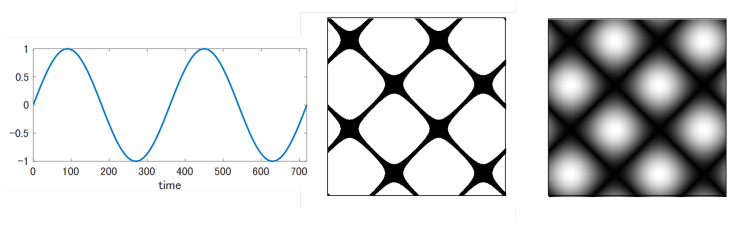
Recurrence plots for sine wave: left: binary image, right: grayscale image.

The RPCD (Recurrence Plots Compression Distance) draws the RP as a grayscale image rather than a binary image by removing the step function and ϵ in order to leave richer information there. That is, ${\mathrm{RP}}_{i,j}=\left|\right|\vec{x}\left(i\right)-\vec{x}\left(j\right)\left|\right|$. To fit into the grayscale image, ${\mathrm{RP}}_{i,j}$ must be mapped to the range [0, 255]. In the sub-sequence, *m* is always set to 1. As an example, Figure 4 contrasts the grayscale RP with the binary RP for the sine wave. Evidently, the grayscale RP holds more minute information than the binary one.

#### 2.2.2. Ck-1 Distance (Campana-Keogh Distance)

Campana et al. [3] devised the CK-1 distance, which measures the distance between two images *x* and *y* supported by a lossy video compression like MPEG-1. The CK-1 distance between *x* and *y* is defined as Equation (4).

(4) $$\begin{array}{c} \mathrm{CK}1\left(x,y\right)=\frac{C(x|y)+C(y|x)}{C(x|x)+C(y|y)}-1. \end{array}$$

C(x|y) signifies the size of a two-frame video compressed by MPEG-1 in which *y* becomes the first frame and *x* occupies the second frame. In the MPEG-1 encoding, *y* becomes an I picture and *x* becomes a P picture. The CK-1 distance satisfies the symmetry, i.e., CK1(x,y)=CK1(y,x). If x=y, $\mathrm{CK}1\left(x,x\right)=\frac{C(x|x)+C(x|x)}{C(x|x)+C(x|x)}-1=1-1=0$. Next, consider the case when *x* is very dissimilar to *y*. Let C(x) be the file size when *x* is compressed by the intra-frame compression. It this case, it holds that C(x|y)≈C(x)+C(y), because most macroblocks in the second frame *x* are to be compressed by the intra-frame compression, if *x* is quite dissimilar to *y*. In the same way, it also holds C(y|x)≈C(x)+C(y). Therefore, CK1(x,y)≈1, because $\frac{C(x|y)+C(y|x)}{C(x|x)+C(y|y)}\approx \frac{C(x)+C(y)+C(y)+C(x)}{C(x|x)+C(y|y)}-1\approx \frac{2(C(x)+C(y))}{C(x)+C(y)}-1=2-1=1.$ Thus, 0≤CK1(x,y)≤1.

If we hope to apply conventional compression distances except the CK-1, such as NCD, we must convert an image into one dimensional sequence, while sacrificing the spatial two-dimensional information in the image. Therefore, the CK-1 distance is innovative in that it can compare images without discarding spatial information.

#### 2.2.3. Calculation of RPCD

RPCD calculates the distance between two time-series data *A* and *B* in the next way. First, it generates two grayscale recurrence plots $R_{A}$ and $R_{B}$ (i.e., grayscale images) for *A* and *B*. Then, the RPCD between *A* and *B* is determined as the CK-1 distance between $R_{A}$ and $R_{B}$. Many distance-based classifiers including the *k*-NN (*k* nearest neighbor) can be combined with the RPCD to classify time-series data.

The greatest advantage of RPCD is the ease of implementation. Users have only to prepare an MPEG-1 encoder publicly available, e.g., FFmpeg. Because the RPCD is only interested in the file size of compressed videos, the users may simply run the MPEG-1 encoder without hacking the video encoding algorithm to obtain the RPCD between *A* and *B*.

### 2.3. Cross Recurrence Plots Compression Distance (CRPCD)

Michael et al. [8] proposed the Cross Recurrence Plots Compression Distance (CRPCD) that extends RPCD. CRPCD uses the Cross Recurrence Plots (CR) [10] instead of the Recurrence Plots (RP). Whereas the RP draws the self-correlation inside a single time-series data, the CR exhibits the cross-correlation between two time-series data *x* and *y*.

Let $CR_{x,y}$ be the grayscale Cross Recurrence Plots between *x* and *y*. The pixel value ${\mathrm{CR}}_{x,y}\left(i,j\right)$ is determined as

(5) $$\begin{array}{c} {\mathrm{CR}}_{x,y}\left(i,j\right)=\left|\right|\vec{x}\left(i\right)-\vec{y}\left(j\right)\left|\right|,\;\;\;\vec{x}\left(\cdot \right),\vec{y}\left(\cdot \right)\in R^{m},\left\{\begin{array}{c} 1\leq i\leq N\\ 1\leq j\leq N \end{array}\right. \end{array}$$

Here, $\vec{x}\left(i\right)$ and $\vec{y}\left(j\right)$ are the *m*-dimensional vectors that correspond to the *i*-th sub-sequence of *x* and the *j*-th sub-sequence of *y*. The CR can identify the co-occurring pattern between *x* and *y*: If ${\mathrm{CR}}_{x,y}\left(i,j\right)$ takes a very low value, it means that *x* and *y* share a co-occurring pattern that starts at the location *i* in *x* and at the location *j* in *y*.

**Figure 5:**
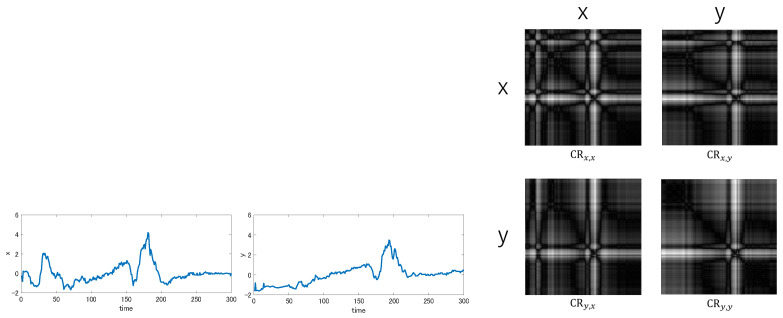
Cross Recurrence Plots between two time-series data *x* and *y*.

For a pair of *x* and *y*, four Cross Recurrent Plots ${\mathrm{CR}}_{x,x}$, ${\mathrm{CR}}_{x,y}$ ${\mathrm{CR}}_{y,x}$ and ${\mathrm{CR}}_{y,y}$ are possible, though ${\mathrm{CR}}_{x,x}$ (${\mathrm{CR}}_{y,y}$) is equivalent to the recurrence plots $R_{x}$ ($R_{y}$, respectively). Figure 5 shows these four Cross Recurrent Plots between a certain pair of time-series data.

The CRPCD evaluates the distance between *x* and *y* by examining all four images ${\mathrm{CR}}_{x,x}$, ${\mathrm{CR}}_{x,y}$ ${\mathrm{CR}}_{y,x}$ and ${\mathrm{CR}}_{y,y}$. The CRPCD aims to enrich the recurrence plots distance with the information on co-occurring patterns. For this purpose, it introduces the CK-2 distance that modifies the CK-1 distance so that all four images may be taken into account. Equation (6) defines the CK-2 distance. Because this formula requires eight two-frame videos, the CK-2 distance spends twice as long time as the CK-1 distance.

(6) $$\begin{array}{c} \mathrm{CK}2\left(x,y\right)=\frac{C\left({\mathrm{CR}}_{x,x}|{\mathrm{CR}}_{x,y}\right)+C\left({\mathrm{CR}}_{x,y}|{\mathrm{CR}}_{x,x}\right)+C\left({\mathrm{CR}}_{y,y}|{\mathrm{CR}}_{y,x}\right)+C\left({\mathrm{CR}}_{y,x}|{\mathrm{CR}}_{y,y}\right)}{C\left({\mathrm{CR}}_{x,x}|{\mathrm{CR}}_{x,x}\right)+C\left({\mathrm{CR}}_{x,y}|{\mathrm{CR}}_{x,y}\right)+C\left({\mathrm{CR}}_{y,x}|{\mathrm{CR}}_{y,x}\right)+C\left({\mathrm{CR}}_{y,y}|{\mathrm{CR}}_{y,y}\right)}-1. \end{array}$$

### 2.4. Time Series Classification

We review previous works on time-series classification, focusing mainly on the methods based on the RP. As for the recent trends of recurrence plots, refer to the comprehensive survey paper [11]. The algorithms to classify time-series data are roughly categorized into two types: (1) classical methods and (2) methods supported by Deep Neural Networks.

The classical methods are divided into two types: (A) distance-based methods and (B) feature-based methods. The distance-based method measures the distance between time-series data to judge whether they enter the same class. Dynamic Time Warping [12] and its extensions [13,14] are representative distance measures for time-series data. Of course, the compression distance is a kind of distance-based method. The feature-based method extracts a group of features from given time-series data. The so-called BoF (Bag-of-Features) belongs to it. Bag of temporal-SIFT-Words [15] adapts the SIFT descriptor to one-dimensional time series and extracts local features densely. Bag of Recurrence-Patterns [16] relies on the RP. By representing time-series data as an image, the SIFT descriptor for images can be migrated to handle time-series data almost as it is. COTE [17,18] is an ensemble method, which integrates 35 classifiers.

Along with the success of CNN (Convolutional Neural Network) in image recognition/classification tasks, the RP has attracted much attention, because we may borrow the strength of the CNN in classifying time-series data, after they are converted to RP images. Hatami et al. [19] attempted to classify the RP images on the CNN architecture early on. Nakano et al. [20] paid attention to the symmetric nature of RP and embedded different information to the half of RPs. Zhang et al. [21] proposed multi-scale signed recurrence plots that retain upward/downward trends and the scale of images. The approach to combine the RP with the CNN is actively applied to medical diagnoses such as Alzheimer’s disease analysis [22] and the detection of Arrhythmia from a given ECG data [23].

## 3. Analysis of RPCD

Although RPCD classifies time-series data quite accurately, its implementation details are omitted in [5]. For instance, we can neither know which MPEG-1 encoder to use nor the settings for the MPEG-1 encoding. Hence, we implemented RPCD by ourselves and investigated its characteristics. As an MPEG-1 video encoder, we select the most famous free universal media converter FFmpeg Version 3.4 [6]. This section explains the interesting insights acquired through our analysis. Although this paper treats only time-series data, our analysis in this section does not explicitly presume time-series data. Therefore, our insights will also be valid for the CK-1 distance that is more primitive than the RPCD. Because the CK-1 distance has been used to detect concept drifts recently [7], the scope of our insights is not limited to time-series classification.

We would state the next two insights below.

- We must specify the quality parameter explicitly for the FFmpeg encoder. This quality parameter must be consistent for the whole dataset to be analyzed.
- The optimal quality parameter varies greatly per dataset.

Let us explain the first one. When the FFmpeg encodes a video with MPEG-1, users may alter the quality parameter by specifying the “q” option. It controls the next two items.

1. The quality scale qs that decides the degree to quantize the DCT coefficients.
2. The range to search motion vectors for the inter-frame prediction. By increasing *q*, the search range narrows down and shrinks the norm of motion vectors.

The readers may consider that it is natural to specify the quality parameter *q* in the MPEG-1 encoding, as we already mentioned qs in Section 2.1. However, in practice, standard FFmpeg users rarely specify *q*. Instead, they specify the target bit rate and expect FFmpeg to adapt the quality scale to the target bit rate. In this mode, FFmpeg applies different quality scales to different video frames, so that the file size of the compressed video is untrustworthy to evaluate the similarity between images objectively. Therefore, we must fix the quality parameter to some constant in classifying a time-series dataset. We claim that, if standard users run the FFmpeg in their familiar way, they cannot operate the RPCD correctly. We believe this is the first trap that they may fall into.

### 3.1. Effect of Quality Parameter

Now, we have understood that the quality parameter should be fixed to some constant. However, to which value should the quality parameter be set for time-series classification? In fact, RPCD and CRPCD were proposed without referring to the quality parameter. As far as we know, only Campana et al. [3] who developed the CK-1 distance wrote about the quality scale. However, they treated natural texture images and only described that they selected large quantized scales to ignore subtle differences caused by noise. Unfortunately, this heuristic rule fails for several time-series dataset.

**Figure 6:**
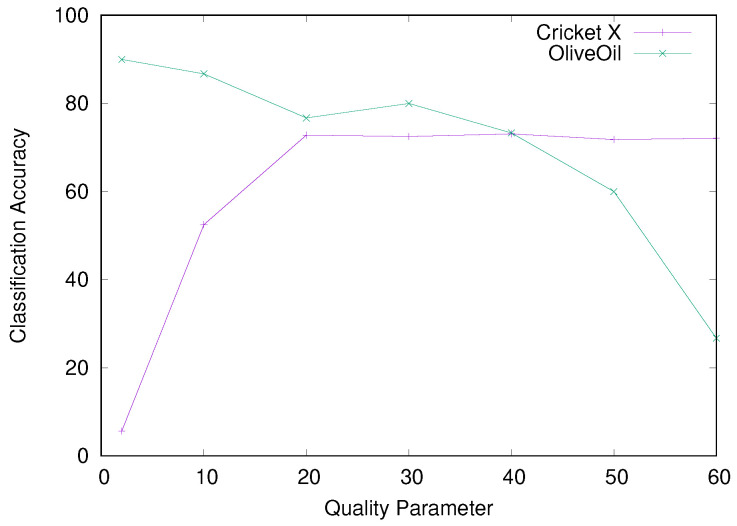
Classification accuracy for various *q* values.

Experimentally, the effect of the quality parameter changes greatly for different dataset: For some dataset, small parameters increase the classification error extremely, whereas large parameters do so for other dataset. Especially, the optimal value for one dataset is occasionally beaten by a naive random classifier for another dataset: Figure 6 presents the classification accuracy for the two dataset “Cricket X” and “OliveOil” from the UCR Time Series Classification Archive [24]. The Cricket X accompanies 12 classes, while the number of classes equals 4 for the OliveOil. We integrate the RPCD with the Nearest Neighbor (NN) classifier. We refer to this classifier as the RPCD-NN. We select the quality parameter from a set of figures {2, 10, 20, 30, 40, 50, 60}.

Remarkably, the Cricket X yielded the exact opposite result to the OliveOil. As *q* becomes greater, RPCD behaves better for the Cricket X and deteriorates for the OliveOil. Especially for q=2, which works best for the OliveOil, the classification accuracy degrades to 5.64% for the Cricket X. Thus, the RPCD-NN becomes inferior to the random classifier for 12 classes. Similarly, for q=60, which works well for the Cricket X, the RPCD-NN only achieves an accuracy of 26.67% for the OliveOil, that is comparable to the random classifier for 4 classes whose expected accuracy equals 25%.

Section 3.2 and Section 3.3 explain why RPCD behaves quite awfully, unless *q* is chosen adequately.

### 3.2. The Case When Small *Q* Values Trouble RPCD

For the Cricket X, the RPCD-NN operates awfully for q=2. Our experiments observed the same phenomenon also for 5 out of the 27 datasets. Table 1 shows the information on the Cricket X.

When q=2, the RPCD-NN is defeated even by the random classifier for the Cricket X. By investigating the reason, we noticed that a few dominant training data become the nearest neighbor for many test data. Concretely, only one training data becomes the nearest neighbor for 192 test data. This result means that the RPCD-NN categorized more than half of the test data to the same class, which evidently includes many mistakes. Table 2 displays the top-3 training data that are selected as the nearest neighbor most frequently.

Such dominant training data have common features. Let *y* be a dominant training data. In most cases, *y* satisfies Property 1 below. To simplify the exposition, we denote the RP of *y*, that is, R${}_{y}$ by the same symbol *y* in the rest of Section 3.

**Property 1:**
  - The compressed video that concatenates *y* twice has a rather large file size.
  - In the compressed video, the second frame occupies a relatively large volume.

Formally, let y|y present the video that concatenates *y* twice. $C_{1}\left(y|y\right)$ and $C_{2}\left(y|y\right)$ represent the size of the first frame and the second frame in y|y after the compression. Obviously, $C\left(y|y\right)=C_{1}\left(y|y\right)+C_{2}\left(y|y\right)$. As the first frame becomes an I picture and the second frame becomes a P picture in the MPEG-1 encoding, the intra-frame compression decides $C_{1}\left(y|y\right)$, while $C_{2}\left(y|y\right)$ is related to the inter-frame prediction. Now, we may rewrite Property 1 as Property 2.

**Property 2:**
  - C(y|y) is rather large and the ratio $\frac{C_{2}\left(y|y\right)}{C(y|y)}$ is also large.

In Table 2, the third column shows $\frac{C_{2}\left(y|y\right)}{C(y|y)}$ and the fourth column displays the rank of $\frac{C_{2}\left(y|y\right)}{C(y|y)}$ in the 390 training data. Thus, Table 2 tells that, for q=2, the top-3 dominant training data are ranked first, second and fourth regarding the size of $\frac{C_{2}\left(y|y\right)}{C(y|y)}$.

Why does $\frac{C_{2}\left(y|y\right)}{C(y|y)}$ become large for some training data only? This phenomenon is attributed to the lossy compression in MPEG-1. Because the first and second frames are the same in the video y|y, one may think reasonably that $C_{2}\left(y|y\right)\approx 0$, after the inter-frame prediction compresses the second frame. However, this is wrong. Actually, the inter-frame prediction compares the second frame *y* with the quantized first frame, say $y^{\prime }$, compressed by the intra-frame compression. Thus, when $y^{\prime }$ is well-compressed, the difference $y-y^{\prime }$ between *y* and $y^{\prime }$ enlarges. Because $y-y^{\prime }$ cannot be compressed enough for a small *q* value, $\frac{C_{2}\left(y|y\right)}{C(y|y)}$ grows large.

On the other hand, when *q* is bigger such as q=20, the intra-frame compression makes $y-y^{\prime }$ bigger by compressing the first frame more aggressively as compared with the case q=2. Nonetheless, $\frac{C_{2}\left(y|y\right)}{C(y|y)}$ lessens, because the inter-frame prediction quantizes $y-y^{\prime }$ more intensely. As a result, dominant training data vanish for q=20. Table 3 displays the top-3 training data that are selected as the nearest neighbor most frequently for q=20: Even the top-1 training data are selected as the nearest neighbor at most 9 times. You can also see that the magnitude of $\frac{C_{2}\left(y|y\right)}{C(y|y)}$ has become moderate. For q=20, the RPCD-NN improves the classification accuracy to 72.82% by excluding the dominant training data.

#### Why Property 2 Generates Dominant Training Data?

In the last, we explain the mechanism that a training data *y* with Property 2 tends to become the nearest neighbor for many test data. Let *x* be a test data.

By separating the compressed video into the first frame and the second frame, we may rewrite CK1(x,y) with Equation (7).

(7) $$\begin{array}{ccc} \mathrm{CK}1(x,y) & = & \frac{C(x|y)+C(y|x)}{C(x|x)+C(y|y)}-1\\ & = & \frac{C_{1}\left(x|y\right)+C_{2}\left(x|y\right)+C_{1}\left(y|x\right)+C_{2}\left(y|x\right)}{C(x|x)+C(y|y)}-1\\ & = & \frac{C_{1}\left(x|y\right)+C_{2}\left(x|y\right)+C_{1}\left(y|x\right)+C_{2}\left(y|x\right)}{C(x|x)+C(y|y)}\\ & & -\frac{C_{1}\left(x|x\right)+C_{2}\left(x|x\right)+C_{1}\left(y|y\right)+C_{2}\left(y|y\right)}{C(x|x)+C(y|y)} \end{array}$$

Here, we have $C_{1}\left(x|y\right)=C_{1}\left(y|y\right)$, because both of them correspond to the file size of the first frame *y* compressed by the intra-frame compression. In the same way, $C_{1}\left(y|x\right)=C_{1}\left(x|x\right)$. Therefore, Equation (7) is equivalent to Equation (8) below.

(8) $$\begin{array}{c} \mathrm{CK}1\left(x,y\right)=\frac{C_{2}\left(x|y\right)+C_{2}\left(y|x\right)}{C(x|x)+C(y|y)}-\frac{C_{2}\left(x|x\right)+C_{2}\left(y|y\right)}{C(x|x)+C(y|y)}. \end{array}$$

In the next, when the test data are classified with the NN, C(x|x) and $C_{2}\left(x|x\right)$ may be regarded as a constant α and β, respectively. Thus, Equation (8) is converted into Equation (9).

(9) $$\begin{array}{c} \mathrm{CK}1\left(x,y\right)=\frac{C_{2}\left(x|y\right)+C_{2}\left(y|x\right)}{\alpha +C(y|y)}-\frac{\beta +C_{2}\left(y|y\right)}{\alpha +C(y|y)} \end{array}$$

In Equation (9), the first term contains $C_{2}\left(x|y\right)$ and $C_{2}\left(y|x\right)$ and surely reflects the similarity between *x* and *y*. On the other hand, the second term has nothing to do with the similarity between *x* and *y* and might disturb the similarity evaluation. Particularly, if $\frac{C_{2}\left(y|y\right)}{C(y|y)}$ is big and C(y|y) is large relative to α, the second term reduces CK1(x,y) unfairly irrespective of how *y* is similar to *x*. In this way, if the training data *y* satisfy Property 2, *y* can become the nearest neighbors for many test data.

### 3.3. The Case When Large *Q* Values Trouble RPCD

**Figure 7:**
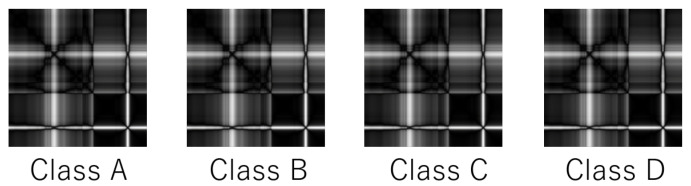
Recurrence plots for different classes in OliveOil.

The RPCD-NN suffers for the OliveOil dataset, when *q* takes a large value. In a dataset like OliveOil, different time-series data generate visually similar RPs, even if they belong to different classes. See Figure 7 as an example. To distinguish these four classes, we have to detect the slight difference among their RPs. However, when *q* is large, the quantization destroys the slight difference and disables fine-grained classification.

Formally, suppose that *x* and *y* are two time-series data from two different classes. If their recurrence plots *x* and *y* are quite similar and *q* is large, the inter-frame prediction quantizes all the 64 DCT coefficients to 0 for the second frame *x* in the video x|y. Thus, $C_{2}\left(x|y\right)\approx C_{2}\left(x|x\right)$. Similarity, $C_{2}\left(y|x\right)\approx C_{2}\left(y|y\right)$. Thus, it holds for any *x* and *y* that

(10) $$\begin{array}{ccc} \mathrm{CK}1(x,y) & = & \frac{C_{2}\left(x|y\right)+C_{2}\left(y|x\right)}{C(x|x)+C(y|y)}-\frac{C_{2}\left(x|x\right)+C_{2}\left(y|y\right)}{C(x|x)+C(y|y)}\;(\mathrm{According}\mathrm{to}\mathrm{Equation}\;(8))\\ & = & \frac{C_{2}\left(x|y\right)-C_{2}\left(x|x\right)+C_{2}\left(y|x\right)-C_{2}\left(y|y\right)}{C(x|x)+C(y|y)}\approx 0. \end{array}$$

Under this environment, the RPCD-NN malfunctions and outputs many classification errors.

## 4. Proposed Methods

Section 3 tells that the quality parameter *q* affects the performance of RPCD seriously. Therefore, we should choose *q* adaptively to the given dataset. On the other hand, Section 3 also implies that there exists no universal method, because the two datasets revealed the exact opposite tendency. Thus, we propose to rely on the validation data to learn *q* that works reasonably for the dataset at hand. Our method is named qRPCD.

qRPCD classifies every training data with the leave-one-out cross-validation (LOOCV) and identifies the optimal *q* value that achieved the highest average classification accuracy, where the average is taken over all the validation data. To classify a TEST data, qRPCD specifies the learned optimal *q* value and executes the RPCD. We remark that we preferred the LOOCV to the *k*-fold cross validation, simply because some datasets in the UCR Time Series Classification Archive prepare a small number of training data. For example, the SonyAIBORobot Surface dataset in the archive holds only 20 training data. In case the target dataset has plenty of training data, the *k*-fold cross validation will be more preferable to LOOCV. In such a situation, the LOOCV is too heavy to execute, because it must compute the RPCD n×(n−1)=n(n−1) times for every *q* value, where *n* represents the number of training data.

**Figure 8:**
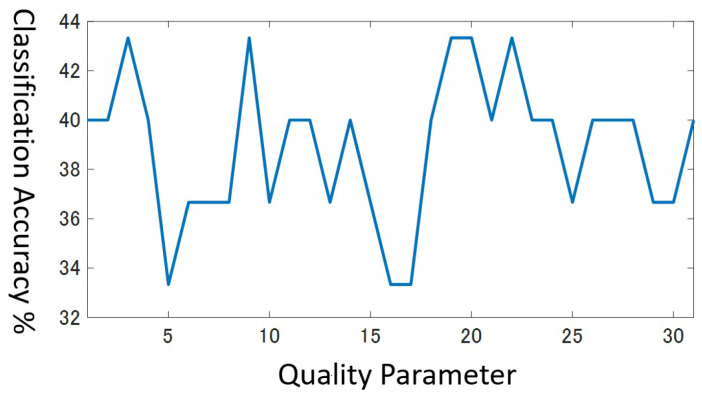
Accuracy for validation data for Beef dataset.

In the cross validation, we search the optimal value in the range 1≤q≤31. However, in practice, multiple values in the range often attain the same highest accuracy simultaneously, especially if the dataset has a few training data. For example, Figure 8 shows the classification accuracy in the range 1≤q≤31 for the validation data in the beef dataset that holds only 30 training data. You can see the highest value corresponds to several *q* values, that is, q=3,9,19,20,22. It is no wonder that there exist multiple peaks in this graph for the next reasons.

- The minimum resolution of accuracy is $\frac{1}{30}=3.3\%$ at the best, when the number of training data is as small as 30.
- Different class pairs may have a different optimal resolution, i.e., video quality to distinguish them.

Which one of the *q* values should we select? Considering that the results with the small-size validation data are unlikely to be solid enough, we desire to choose the least risky value. As for the beef dataset, we regard q=9 as more risky than q=20, because the classification accuracy drops abruptly just by changing *q* slightly. In order to seek a stable *q* value, we assume that a field of classification accuracy form in the range [1, 31] and apply the smoothing filter in Equation (11) to the field three times.

(11) $$\begin{array}{c} y\left(q\right)=\frac{1}{4}\left\{x\left(q-1\right)+2x\left(q\right)+x\left(q+1\right)\right\} \end{array}$$

**Figure 9:**
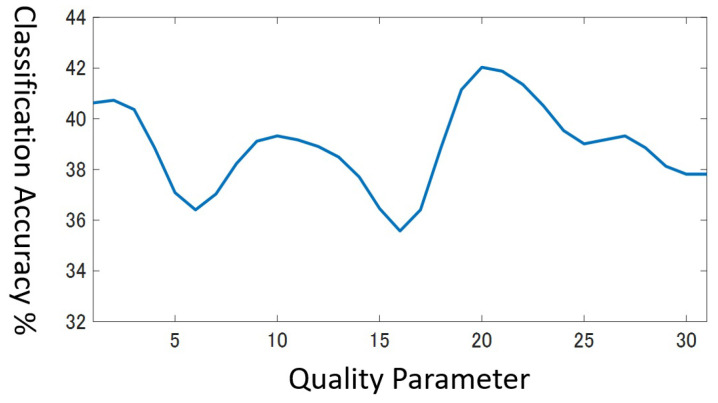
Smoothed accuracy for validation data for Beef dataset.

In Equation (11), x(q) and y(q) present the classification accuracy for *q* before and after applying the smoothing filter, respectively. Finally, we choose the *q* value whose smoothed classification accuracy grows the highest. Figure 9 draws the smoothed classification accuracy for the beef dataset. Now, we can choose the unique best value as q=20.

## 5. Results

We conduct experiments on time-series classification. First, we compare qRPCD with two previous compression distance RPCD and CRPCD based on RPs. Recall that every compression distance is combined with the NN classifier. We also compare qRPCD with the Bag-of-Recurrent-Patterns (BoRP) [16] that is more recent than RPCD and CRPCD. BoRP is the latest feature-based method that exploits the RP.

The experiments involve 27 dataset from the UCR Time Series Classification Archive. For these dataset, the training data and the test data are officially partitioned. These dataset are categorized into the next five types: (1) Image. Many datasets of this type represent the outline of objects as time-series data. (2) Sensor. (3) Motion that expresses a trajectory of human motion as a time-series data. (4) Spectro that collects spectral analysis data for foods. (5) ECG (Electrocardiogram). Table 4 shows the types of the 27 datasets.

### 5.1. Comparison with Previous Recurrence-Plots Compression Distances

Table 5 compares qRPCD with RPCD and CRPCD in terms of classification accuracy. For RPCD and CRPCD, we cite their accuracy rates from their original papers, that is, ref. [5] for RPCD and [8] for CRPCD. As for our qRPCD, we also list the learned *q* value in the second rightmost column. For each dataset, the most accurate compression distance is marked by writing its accuracy rate in bold fonts.

qRPCD outperforms the original RPCD by about 4% and the CRPCD by about 5% on average. It achieves the highest accuracy including ties for 20 out of the 27 datasets. From this result, we conclude that learning the optimal quality parameter with the validation data is quite effective to enhance the RPCD.

However, qRPCD is defeated by the original RPCD for the two dataset “DiatomSizeReduction” and “Lightning7”. We infer the reason as follows: These two dataset prepare a small number of training data, so that the cross validation fails to identify the adequate *q* value. In fact, the number of training data is only 16 for DiatomSizeReduction and 70 for Lightning7.

All of the qRPCD, RPCD and CRPCD express a one-dimensional time-series data with a two-dimensional image, i.e., RP. To access the effectiveness of this strategy, we compare them with a compression distance that directly compresses the one-dimensional series of data. Here we cite the results in [25] that adopted the CDM [26] (Compression-Distance Measure). For two time-series data, *x* and *y*, CDM(x,y) is defined as $\frac{C(y|x)}{C(x)+C(y)}$. The rightmost column in Table 5 summarizes the classification accuracy for the CDM. It shows that the CDM is by far worse than the RP-based methods. It is convincing that the CDM operates poorly: The one-dimensional data compression in CDM only concerns co-occurring words, in other words, co-occurring local features. By contrast, by comparing every pair of time instances at the beginning, the RP additionally extracts other wide-area features spreading over long time periods.

### 5.2. Comparison with Bag-of-Recurrence Patterns (BoRP)

Table 6 compares the classification accuracy of qRPCD with that of BoRP. Again, the accuracy rate for BoRP is cited from the original paper [16]. qRPCD outperforms BoRP by about 2.1% on average. It achieves the highest accuracy including ties for 16 out of the 27 dataset. This result is interpreted as follows: Learning the quality parameter raises the RPCD to a level that surpasses the best classical method based on RPs.

Furthermore, qRPCD works comparably to RP1+ResNet [20]. RP1+ResNet is a CNN-based method developed in 2019. According to [20], its average accuracy for the 27 dataset equals 78.8%, which is smaller than qRPCD. This result is astonishing, since qRPCD needs only one parameter, i.e., much fewer parameters to learn than the CNN.

## 6. Conclusions

This paper studies the RPCD (Recurrence Plots Compression Distance), which measures the similarity between two time-series data by relying on the MPEG-1 video compression. RPCD has a significant advantage that any user can utilize it easily, once they prepare a publicly available MPEG-1 encoder. However, its implementation details have not been disclosed so far, such as which MPEG-1 encoder to use or how to operate the MPEG-1 encoder.

To investigate the characteristics of RPCD, we first implement the RPCD by using the most well-known free MPEG-1 encoder FFmpeg. This paper states the important insights acquired through our analysis as follows. Since our analysis did not presume time-series data explicitly, our insights will hold true also for the more general CK-1 distance.

- We must give a quality parameter *q* manually to the FFMPEG encoder and this parameter value must be fixed for the whole dataset to be analyzed. Because normal users of FFmpeg specify some bit rate and expect the encoder to change the quality parameter dynamically to the bit rate, users who try RPCD for the first time will have trouble without specifying the quality parameter.
- *q* strongly impacts the classification accuracy of the RPCD-NN. The optimal *q* value differs extremely per dataset. For instance, as *q* becomes larger, the classification accuracy increases for some dataset and decreases for another dataset. The performance degradation for inadequate *q* values is caused by the lossy compression in MPEG-1.

Based on the second insight, we propose an extension of RPCD named qRPCD that learns a reasonable *q* value by means of the leave-one-out cross-validation. qRPCD searches a *q* value that achieves the highest classification accuracy for the validation data in the range 1≤q≤31. To cope with the complex situations in which multiple *q* values become the most accurate at the same time, we assume that a field of classification accuracy forms in the range 1≤q≤31 and apply some smoothing filter to the classification accuracy.

Experimentally, qRPCD achieved a higher average classification accuracy than the original RPCD by 4% and than CRPCD by 5% for the 27 dataset from the UCR time-series classification archive. qRPCD is sometimes inferior to the original RPCD, if the dataset arranges only a few training data. Therefore, one future work of this research is to estimate the optimal *q* value more precisely in such cases. Another interesting research direction is to examine if the compression-based pattern recognition founded upon LOSSLESS compression methods such as bzip2 and LZW benefits by learning a key parameter in the same way as qRPCD.

## Figures and Tables

**Table 1:** Cricket X dataset.

# of Test Data	# of Training Data	Data Length	# of Classes
390	390	300	12

**Table 2:** Top 3 dominant training data that are most frequently selected as NN (q=2).

Training Data ID	Selected Times	$\frac{C_{2}\left(y|y\right)}{C(y|y)}$	Rank of $\frac{C_{2}\left(y|y\right)}{C(y|y)}$
135	192	0.2200	1
289	40	0.1986	2
288	37	0.1904	4

**Table 3:** Top 3 training data that are most frequently selected as NN (q=20).

Training Data ID	Selected Times	$\frac{C_{2}\left(y|y\right)}{C(y|y)}$	Rank of $\frac{C_{2}\left(y|y\right)}{C(y|y)}$
146	9	0.0673	23
135	6	0.1064	1
118	6	0.0229	346

**Table 4:** 5 types of dataset.

Image	Sensor	Motion	Spectro	ECG
50words	ItalyPowerDemand	Cricket X	Beef	CinC ECG torso
Adiac	Lightning2	Cricket Y	Coffee	ECG200
DiatomSize	Lightning7	Cricket Z	OliveOil	ECGFiveDays
FISH	SonyAIBORobot	Gun Point		
FaceFour	SonyAIBORobotII	Haptics		
MedicalImages		InlineSkate		
OSULeaf				
SwedishLeaf				
Symbols				
WordsSynonyms				

**Table 5:** Comparison of qRPCD with RPCD and CRPCD.

Dataset	RPCD	CRPCD	qRPCD	*q* for qRPCD	CDM
50words	77.36	78.46	**78.68**	12	N/A
Adiac	61.64	61.38	**71.36**	4	25.58
Beef	**63.33**	46.67	**63.33**	20	40.00
CinC ECG torso	97.90	93.19	**97.97**	10	47.17
Coffee	**100.0**	85.71	**100.0**	1	85.71
Cricket X	70.77	**75.64**	74.87	29	23.08
Cricket Y	73.85	**82.56**	75.13	19	23.08
Cricket Z	70.77	**77.69**	74.62	22	21.28
DiatomSizeReduction	**96.41**	96.08	94.12	1	76.14
ECG200	86.00	88.00	**89.00**	24	73
ECGFiveDays	86.41	80.48	**94.08**	1	64.58
FISH	87.43	76.00	**95.43**	3	41.71
FaceFour	94.32	**95.45**	**95.45**	12	56.82
Gun Point	**100.0**	98.67	**100.0**	1	79.33
Haptics	38.64	41.23	**44.48**	24	29.22
InlineSkate	32.00	35.45	**43.63**	8	21.09
ItalyPowerDemand	84.26	83.77	**94.85**	11	72.11
Lightning2	75.41	**81.97**	75.41	27	67.21
Lightning7	64.38	**69.86**	58.90	31	28.77
MedicalImages	71.05	**71.97**	71.58	21	46.32
OSULeaf	64.46	65.29	**83.06**	1	42.56
OliveOil	83.33	73.33	**90.00**	1	73.33
SonyAIBORobotSurface	79.70	79.70	**85.69**	19	59.57
SonyAIBORobotSurfaceII	84.26	84.47	**86.15**	19	56.98
SwedishLeaf	90.24	88.80	**91.36**	15	34.72
Symbols	90.45	90.05	**97.49**	24	71.06
WordsSynonyms	72.41	73.35	**76.49**	18	23.51
Best	4/27	7/27	**20/27**		0/26
Average	77.66	76.86	**81.60**		49.38

**Table 6:** Comparison with BoRP.

Dataset	BoRP	qRPCD
50words	62.9	**78.7**
Adiac	**77.0**	71.4
Beef	**73.4**	63.3
CinC ECG torso	74.5	**98.0**
Coffee	**100.0**	**100.0**
Cricket X	**100.0**	74.9
Cricket Y	72.6	**75.1**
Cricket Z	**76.3**	74.6
DiatomSizeReduction	90.2	**94.1**
ECG200	**89.2**	89.0
ECGFiveDays	83.1	**94.1**
FISH	**97.2**	95.4
FaceFour	91.0	**95.5**
Gun Point	**100.0**	**100.0**
Haptics	**53.8**	44.5
InlineSkate	38.6	**43.6**
ItalyPowerDemand	94.2	**94.9**
Lightning2	**79.7**	75.4
Lightning7	**74.5**	58.9
MedicalImages	65.2	**71.6**
OSULeaf	**91.9**	83.1
OliveOil	79.2	**90.0**
SonyAIBORobotSurface	84.0	**85.7**
SonyAIBORobotSurfaceII	73.8	**86.2**
SwedishLeaf	**93.2**	91.4
Symbols	95.2	**97.5**
WordsSynonyms	36.3	**76.5**
Best	13/27	**16/27**
Average	79.5	**81.6**

## Data Availability

All the dataset used in our experimentation originate from the UCR Time Series Classification Archive. Refer to https://www.cs.ucr.edu/~eamonn/time_series_data_2018/ to access the dataset archive (accessed on 1 June 2023).
